# Autopsy Prevalence of Tuberculosis and Other Potentially Treatable Infections among Adults with Advanced HIV Enrolled in Out-Patient Care in South Africa

**DOI:** 10.1371/journal.pone.0166158

**Published:** 2016-11-09

**Authors:** Aaron S. Karat, Tanvier Omar, Anne von Gottberg, Mpho Tlali, Violet N. Chihota, Gavin J. Churchyard, Katherine L. Fielding, Suzanne Johnson, Neil A. Martinson, Kerrigan McCarthy, Nicole Wolter, Emily B. Wong, Salome Charalambous, Alison D. Grant

**Affiliations:** 1 TB Centre, London School of Hygiene & Tropical Medicine, London, United Kingdom; 2 Department of Anatomical Pathology, National Health Laboratory Service and University of the Witwatersrand, Johannesburg, South Africa; 3 Centre for Respiratory Diseases and Meningitis, National Institute for Communicable Diseases of the National Health Laboratory Service, Johannesburg, South Africa; 4 School of Pathology, Faculty of Health Sciences, University of the Witwatersrand, Johannesburg, South Africa; 5 The Aurum Institute, Johannesburg, South Africa; 6 School of Public Health, Faculty of Health Sciences, University of the Witwatersrand, Johannesburg, South Africa; 7 Foundation for Professional Development, Pretoria, South Africa; 8 Perinatal HIV Research Unit, and Medical Research Council Soweto Matlosana Collaborating Centre for HIV/AIDS and TB, University of the Witwatersrand, Johannesburg, South Africa; 9 Johns Hopkins University Center for TB Research, Baltimore, Maryland, United States of America; 10 Department of Science and Technology / National Research Foundation Centre of Excellence for Biomedical TB Research, University of the Witwatersrand, Johannesburg, South Africa; 11 Division of Public Health, Surveillance and Response, National Institute for Communicable Disease of the National Health Laboratory Service, Johannesburg, South Africa; 12 Africa Health Research Institute, Durban, South Africa; 13 Division of Infectious Diseases, Massachusetts General Hospital, Boston, Massachusetts, United States of America; 14 University of KwaZulu-Natal, Durban, South Africa; Fundació Institut d’Investigació en Ciències de la Salut Germans Trias i Pujol, Universitat Autònoma de Barcelona, SPAIN

## Abstract

**Background:**

Early mortality among HIV-positive adults starting antiretroviral therapy (ART) remains high in resource-limited settings, with tuberculosis (TB) the leading cause of death. However, current methods to estimate TB-related deaths are inadequate and most autopsy studies do not adequately represent those attending primary health clinics (PHCs). This study aimed to determine the autopsy prevalence of TB and other infections in adults enrolled at South African PHCs in the context of a pragmatic trial of empiric TB treatment (“TB Fast Track”).

**Methods and Findings:**

Adults with CD4 ≤150 cells/μL, not on ART or TB treatment, were enrolled to TB Fast Track and followed up for at least six months. Minimally invasive autopsy (MIA) was conducted as soon as possible after death. Lungs, liver, and spleen were biopsied; blood, CSF, and urine aspirated; and bronchoalveolar lavage fluid obtained. Samples underwent mycobacterial, bacterial, and fungal culture; molecular testing (including Xpert® MTB/RIF); and histological examination. 34 MIAs were conducted: 18 (53%) decedents were female; median age was 39 (interquartile range 33–44) years; 25 (74%) deaths occurred in hospitals; median time from death to MIA was five (IQR 3–6) days. 16/34 (47%) had evidence of TB (14/16 [88%] with extrapulmonary disease; 6/16 [38%] not started on treatment antemortem); 23 (68%) had clinically important bacterial infections; four (12%) cryptococcal disease; three (9%) non-tuberculous mycobacterial disease; and two (6%) *Pneumocystis* pneumonia. Twenty decedents (59%) had evidence of two or more concurrent infections; 9/16 (56%) individuals with TB had evidence of bacterial disease and two (13%) cryptococcal disease.

**Conclusions:**

TB, followed by bacterial infections, were the leading findings at autopsy among adults with advanced HIV enrolled from primary care clinics. To reduce mortality, strategies are needed to identify and direct those at highest risk into a structured pathway that includes expedited investigation and/or treatment of TB and other infections.

## Background

Mortality among HIV-positive adults prior to starting and in the first year of antiretroviral therapy (ART) remains high, particularly among those with advanced disease in resource-constrained settings [1–3]. Pathological autopsy studies involving HIV-positive individuals have consistently found tuberculosis (TB) to be the leading cause of death and the overall prevalence of active TB to be extremely high, much of it undiagnosed antemortem [4–7]. The World Health Organization (WHO) aims to reduce TB deaths by 95% by 2035 [8], but questions remain around current estimates of TB deaths and the methods used to obtain them [9]. Death certification in many parts of sub-Saharan Africa remains sub-optimal [10,11] and the shortcomings of verbal autopsy, a structured interview with the family or carers of the deceased, in classifying HIV-related deaths are well documented [12–14]. Pathological autopsy studies provide the most accurate estimates, but almost all have only included individuals recruited after admission to hospital and do not necessarily represent the broader population who receive the majority of their care from primary health clinics and may die outside of hospitals [15]. Robust data on the prevalence of TB in deceased HIV-positive individuals and more accurate cause of death estimates are essential in measuring progress towards the reduction and eventual elimination of TB-related deaths and in the design of interventions to reduce mortality in this population [16].

Full pathological autopsy with visualisation and sampling of all organs remains the gold standard for assigning cause of death [17,18], but it is expensive, time consuming, and not well accepted by families, who are often required to provide consent [19]. There is growing evidence that minimally invasive autopsy (MIA) can provide useful information relevant to cause of death, particularly with regards to TB and other infectious diseases [20,21]; one study, involving 96 HIV-positive adults in Uganda, compared histology from MIA to that from full autopsy and found that MIA was 71% sensitive and 100% specific in detecting TB [18]. Adding culture and/or other bacteriological modalities to MIA would likely improve its sensitivity in this regard. The acceptance of MIA among recently bereaved families is considerably higher than for full autopsy [5,22]; it is safer, cheaper, and procedures can be conducted outside of hospitals, in private mortuaries or other locations [23]. This study aimed to determine the autopsy prevalence of TB and other treatable infections in adults with advanced HIV disease recruited in public sector primary health care facilities who died after enrolment to a pragmatic trial of empiric TB treatment (“TB Fast Track”) [24].

## Methods

### Study population

The TB Fast Track study, described in detail elsewhere [24], was a cluster-randomised trial of targeted empirical TB treatment enrolling adults with CD4 ≤150 cells/μL, not on TB treatment or ART at the point of enrolment, attending one of twenty-four public sector primary health clinics in three provinces of South Africa. At intervention sites, individuals were assessed for their probability of active TB disease using an experimental algorithm that included the results of point-of-care tests. Those considered ‘high’ probability were started on TB treatment immediately, followed by ART within two weeks; those considered ‘medium’ probability were further investigated; and those considered ‘low’ probability initiated ART immediately. Participants at control sites followed standard clinic procedures. All participants were followed up for at least six months. If the study team became aware of a participant’s death before the funeral had taken place, the family’s permission was sought to undertake MIA. Efforts were made to conduct autopsies as soon as possible after death, but no limits were placed on the time that could elapse between death and autopsy.

### Autopsy procedures

Four organs were targeted for tissue biopsy: liver, spleen, and right and left lungs. Where possible, ultrasound was used to check for liver lesions and pleural effusions and to locate the spleen. Any lesions or effusions found were targeted during sampling. The skin overlying all sampling sites was cleaned using 70% isopropyl alcohol and 10% povidone iodine. Using 18 gauge core-biopsy needles, a minimum of five samples, each approximately 2cm in length, were taken from each site for each of bacterial and fungal microscopy and culture; mycobacterial culture; and histology. Cerebrospinal fluid (CSF) was extracted by insertion of a 22 gauge spinal needle inferior to the occipital base in the direction of the eyes to access the cerebellomedullary cistern. Flocked swabs were inserted into the oro- and naso-pharyngeal cavities and then immersed in PrimeStore media (Longhorn Vaccines and Diagnostics, Bethesda, MD, USA). Blood was aspirated from the right subclavian or internal jugular vein and a urinary catheter was used to collect residual urine. Modified bronchoalveolar lavage (BAL) was conducted by instilling 80-120ml of 0.9% saline through a horizontal incision of the cricothyroid membrane using a 16 gauge nasogastric tube inserted into the trachea in the direction of the lungs. Enlarged lymph nodes and skin lesions in areas other than the face were sampled by needle biopsy or scalpel excision, respectively.

As far as possible, MIA was conducted by a single, clinically-trained operator. If the primary operator was unavailable, limited MIA, without ultrasound or any attempt to obtain blood, urine, or BAL, was conducted by a non-clinical operator.

### Laboratory procedures

All tissue samples, as well as CSF, urine, and BAL were cultured for mycobacteria using Mycobacterial Growth Indicator Tube (MGIT) culture (Becton Dickinson Microbiology Systems, Cockeysville, MD, USA). Culture-positive samples underwent microscopy with Ziehl-Neelsen (ZN) staining and polymerase chain reaction (PCR) testing for speciation and drug resistance (Hain Lifescience GmbH, Germany). Xpert® MTB/RIF (Cepheid, Sunnyvale, CA, USA) and microscopy of immunofluorescence-stained slides for *Pneumocystis jirovecii* were performed on BAL samples. Tissue samples, as well as CSF, blood, and BAL, underwent microscopy, Gram staining, and aerobic culture for bacteria and fungi. Real-time PCR assays were used to detect *Streptococcus pneumoniae*, *Neisseria meningitidis*, and *Haemophilus influenzae* in CSF and blood [25,26]; and *Legionella* spp., *Mycoplasma pneumoniae*, *Chlamydia pneumoniae* [27], and *Bordetella* spp. [28] in naso-/oro-pharyngeal and BAL specimens. Naso-/oro-pharyngeal and BAL specimens were further tested by multiplex real-time reverse transcription PCR for parainfluenza viruses 1–3, adenovirus, enterovirus, human metapneumovirus, respiratory syncytial virus, rhinovirus, influenza A virus, and influenza B virus [29]. CSF was tested for the presence of parvovirus B19, cytomegalovirus (CMV), Epstein Barr virus (EBV), herpes simplex virus 1–2, human herpes viruses 6 and 7, and parechoviruses (FTD Neuro 9 assay, Fast-track diagnostics Ltd., Malta). Urine was tested for *Legionella pneumophila* serogroup 1 and *S*. *pneumoniae* antigen (BinaxNOW, Alere, Waltham, MA, USA). Histological examination was carried out on all tissue samples, using ZN and Grocott stains for the identification of mycobacteria and fungi, respectively. Other stains and PCR procedures were performed as necessary, based on pathologist assessment.

### Ethics

Separate approvals for the autopsy sub-study were obtained from the Research Ethics Committees of the London School of Hygiene & Tropical Medicine and The University of the Witwatersrand. Beginning in August 2013, participants in TB Fast Track were asked to give written informed consent for MIA if they should die during the parent study. Declining permission to take part in the autopsy sub-study did not affect their participation in TB Fast Track or their routine health care. If a participant who had given their written consent died during follow-up, verbal agreement from the next of kin was obtained to proceed with MIA. For participants who were enrolled to TB Fast Track prior to August 2013 and died during follow-up, formal written consent to undertake MIA was sought from the next of kin.

### Data interpretation and statistical analysis

Data were entered into EpiData v3.1 (The EpiData Association, Odense, Denmark) and analysed using Stata v14 (StataCorp, College Station, TX, USA). Baseline demographics were compared using chi-square or Kruskal-Wallis tests, as appropriate. Analysis was conducted in order to estimate the autopsy prevalence of identified pathogens only; no inference was made as to possible cause(s) of death, which will be reported elsewhere. Microscopy and culture results were interpreted by experienced microbiologists who reviewed the data and designated, for each decedent, which organisms were considered ‘artefact’ and which ‘pathogenic’. For all decedents, *Enterococcus* spp., *Enterobacter* spp., *E*. *coli*, *Proteus* spp., *Bacillus* spp., coagulase negative staphylococci, and *Viridans streptococci* were considered artefact. Additionally, Gram-negative organisms isolated only from the spleen of a decedent were considered artefact due to the high likelihood of contamination from abdominal viscera.

## Results

### Consent, demographics, and samples obtained

From December 2012 to December 2014, a total of 3022 individuals were enrolled into the TB Fast Track study: 626 (20.7%) were enrolled prior to autopsy sub-study initiation and were not asked to consent at the point of enrolment. Of the remaining 2396 individuals, 1675 (69.9%) consented to autopsy at enrolment. A total of 364 TB Fast Track participants died after enrolment to the study, 285 (78%) within six months. Seventy-five of 364 (21%) deaths occurred prior to sub-study initiation and 229 (63%) were ascertained after burial, leaving 60 (16%) individuals for whom death was ascertained in time for MIA. Of these 60, 14 (23%) had already declined to participate and for three (5%) no family member could be contacted. Between October 2013 and June 2015, 43 families were approached in person for permission to conduct an autopsy: 36 (84%) gave their consent. Two autopsies did not proceed due to refusal by the mortuary; 34 autopsies were therefore conducted.

Decedents included in the MIA study are described in Table 1. Just over half the decedents (18/34 [53%]) were female; median age was 39 (interquartile range [IQR] 33–44) years; all were Black African; 32 (94%) were South African, with one (3%) from each of Lesotho and Mozambique; 13 (38%) individuals had completed South African grade 12 (high school) or equivalent; 17 (50%) were in some form of employment at the point of enrolment; and, of the 24 individuals able to estimate their household income at enrolment, 15 (63%) reported it to be less than R2000 (~USD150) per month. The majority of individuals died in hospitals (25/34 [74%]), the remainder died in the community; median time from enrolment to death was 60 (IQR 21–175) days; and 28 (82%) individuals died within six months of enrolment. The median CD4 count at enrolment was 34 (IQR 17–66) cells/μL. Twenty (59%) decedents were started on TB treatment a median 60 (IQR 26–175) days prior to death: 15/20 (75%) were enrolled to the intervention arm of the TB Fast Track study and 12 (80%) of these were started on TB treatment based on the study algorithm. Twenty-five (74%) decedents were initiated on ART a median 74 (IQR 30–126) days before death; 28 (82%) started co-trimoxazole prophylaxis at least one week before death; and one (3%) isoniazid preventative therapy at least one week before death. A comparison of baseline characteristics between individuals who had an MIA conducted (n = 34) and those who died within six months of enrolment but did not have an MIA (n = 259) showed only one important difference, in the proportion who started TB treatment between enrolment and death (59% in MIA group and 31% in group with no MIA; *p* = 0.001).

**Table 1:** Baseline characteristics of deceased TB Fast Track participants: MIA conducted (n = 34) vs no MIA (n = 259).

Characteristics		MIA conducted (n = 34) n (%) or median (IQR)	No MIA conducted (n = 259) n (%) or median (IQR)	*p* value (Chi^2^ or Kruskal-Wallis)
**Demographics and past medical history**				
	Female	18 (53)	133 (51)	0.86
	Age (years)	39 (33–44)	39 (34–46)	0.57
	CD4 count at enrolment (cells/μL)	34 (17–66)	45 (21–88)	0.21
	Black African	34 (100)	257 (99)	0.61
	South African	32 (94)	242 (93)	0.88
	Enrolled in a peri-urban area	23 (68)	187 (72)	0.58
	Completed grade 12	13 (38)	82 (32)	0.44
	Employed at time of enrolment	17 (50)	106 (41)	0.31
	Household income of ≤R2000 per month*	15 (63) (n = 24)	90 (47) (n = 190)	0.16
	Previously diagnosed with chronic illness**	5 (15)	34 (13)	0.80
	Smoking history	7 (21) (n = 33)	51 (20) (n = 257)	0.85
	Previous treatment for TB	6 (18)	27 (10)	0.21
**Symptoms at enrolment**				
	Cough	14 (41)	127 (49)	0.39
	Night sweats	11 (32)	85 (33)	0.96
	Fever	11 (32)	73 (28)	0.61
	Weight loss	28 (88) (n = 32)	205 (84) (n = 244)	0.61
**Assessment at enrolment**				
	Body-mass index (kg/m^2^)	20.2 (18.7–23.2)	20.2 (17.7–23.6)	0.48
	Positive urine LAM (point of care) †	10 (29)	74 (29)	0.92
**Between enrolment and death**				
	Started TB treatment	20 (59)	79 (31)	0.001
	Started ART	25 (74)	153 (59)	0.10
	Time from enrolment to death (days)	60 (21–175)	60 (31–113)	0.72

*Ten decedents in the MIA group and 69 in the no MIA group did not know their average household monthly income.

**Chronic illness included hypertension, diabetes, asthma, epilepsy, cancer, and cardiac, renal, and chronic lung disease.

†Samples that elicited one or more visible lines on the LAM test strip were considered positive.

ART: antiretroviral therapy; IQR: interquartile range; LAM: lipoarabinomannan; MIA: minimally-invasive autopsy; TB: tuberculosis

The primary operator conducted 31/34 (91%) MIA; median time from death to MIA was five (IQR 3–6) days; and 33/34 (97%) MIA were conducted within 10 days of death. Measured by histological examination, overall success rates in obtaining samples varied by site: 33/34 (97%) attempted lung biopsies were successful, compared to 30/34 (88%) liver and 21/34 (62%) splenic biopsies; 30/31 (97%), and 30/32 (94%) attempts to obtain BAL and CSF, respectively, were successful (S1 Table).

### Tuberculosis

**Fig 1:**
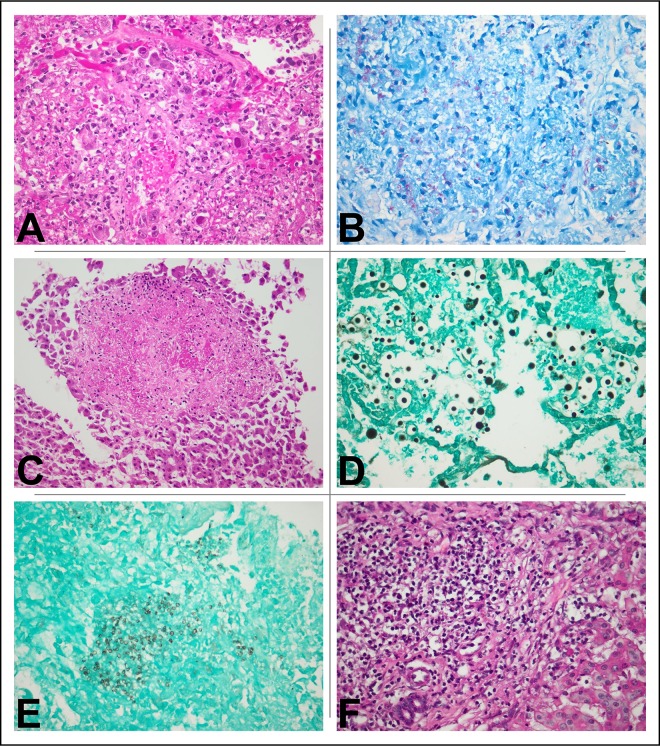
Six examples of histological changes observed in MIA tissue samples (n = 4). Panels (A), (B), and (C) demonstrate mycobacterial and CMV disease in the lung and liver of decedent 08: (A) necrotising, intra-alveolar granuloma with superadded CMV nuclear inclusions (lung; H&E x40); (B) numerous acid-fast bacilli (lung; ZN x40); and (C) mycobacterial granuloma (liver; H&E x20). Panel (D) demonstrates polymorphous cryptococcal yeasts in alveolar spaces (decedent 19; lung; Grocott’s methenamine silver x40). Panel (E) illustrates the characteristic small, crescenteric, yeast-like fungi of *Pneumocystis jirovecii* pneumonia (decedent 11; lung; Grocott’s methenamine silver x40). Panel (F) shows active hepatitis B virus infection (decedent 34; liver; H&E x40). CMV: cytomegalovirus; H&E: haematoxylin and eosin; MIA: minimally invasive autopsy; ZN: Ziehl-Neelsen

Evidence of TB disease was found in 16/34 (47%) decedents (Table 2), of whom 12 (75%) had evidence on histology (Fig 1); 11 (69%) on culture; five (31%) on testing with Xpert® MTB/RIF; and eight (50%) on histology plus bacteriology. Almost all (14/16 [88%]) decedents with evidence of TB had extrapulmonary disease; 3/14 (21%) had no evidence of pulmonary disease. BAL specimens provided the highest yield for culture (seven positive), followed by CSF and splenic tissue (five each), and lung and liver tissue (four each; S1 Table). Twelve decedents had histological evidence of TB: four (33%) in all of lungs, liver, and spleen; two (17%) in lungs and liver only; two (17%) in liver and spleen only; and two (17%) in lungs alone. A total of 164 samples underwent mycobacterial culture: 37 (23%) were positive and 13 (8%) could not be interpreted due to bacterial contamination. Of the 16 individuals with autopsy evidence of TB, 10 (63%) were started on TB treatment a median 56 (IQR 40–173) days before death; 14 (88%) received ART, initiated a median 65 (IQR 30–126) days before death; and nine (56%) were initiated on both TB treatment and ART. Six (38%) individuals with autopsy evidence of TB were not started on TB treatment antemortem, five of whom were enrolled to the control arm of TB Fast Track.

**Table 2:** Combined histopathological and microbiological findings; participants with evidence of each at autopsy; and demographics for select groups (n = 34).

Pathogen			Decedents with autopsy evidence of		Age at death (years) Median (IQR or range*)	CD4 count at enrolment (cells/μL) Median (IQR or range*)	Time from enrolment to death (days) Median (IQR or range*)
	Major disease category		Major disease category n (%/34)	Disease sub-category n (row %)			
		Disease sub-category					
**All decedents**			**-**	**-**	**39 (33–44)**	**34 (17–66)**	**60 (21–175)**
**Mycobacteria**							
	**Tuberculosis**		**16 (47)**	-	38 (33–43)	44 (21–60)	63 (40–152)
		Pulmonary only		2 (13)			
		Extrapulmonary only		3 (19)			
		Pulmonary & extrapulmonary		11 (69)			
		Rifampicin-resistant		2 (13)			
	**Non-tuberculous mycobacteria**		**3 (9)**		45 (27–56)	2 (2–7)	33 (21–324)
		Disseminated		3 (100)			
**Bacteria†**							
	**Any bacterial infection**		**23 (68)**		40 (34–45)	33 (10–75)	57 (21–184)
	**Pneumonia**		**11 (32)**		41 (38–54)	33 (17–36)	33 (14–285)
		Gram-negative		6			
		Gram-positive		5			
		Organism unclear		1			
	**Bacteria in CSF**		**4 (12)**		40 (31–54)	106 (66–132)	82 (8–285)
		*S*. *pneumoniae*		3			
		*S*. *aureus*		1			
		*Salmonella* sp.		1			
	**Bacteraemia**		**3 (9)**		33 (31–36)	106 (29–132)	100 (8–184)
		*H*. *influenzae*		1			
		*K*. *pneumoniae*		1			
		*Salmonella* sp.		1			
		*S*. *aureus*		1			
		*S*. *pneumoniae*		1			
**Fungi & viruses‡**							
	**Pneumonitis**		**4 (12)**		38 (29–44)	86 (17–106)	67 (14–304)
	**Cryptococcal disease**		**4 (12)**		40 (29–46)	8 (1–56)	53 (10–373)
		Disseminated		2 (50)			
	**Pneumocystis pneumonia**		**2 (6)**		40 (39–41)	41 (3–78)	133 (84–182)
	**CMV disease**		**2 (6)**		33 (33–34)	20 (10–29)	106 (28–184)
		Disseminated		2 (100)			
**Other/non-infectious**							
	**Hepatic**		**18 (53)**		40 (34–45)	29 (17–56)	91 (33–184)
		Steatosis		12 (67)			
		Non-specific portal triaditis		6 (33)			
		Chronic hepatitis? viral		4 (22)			
		Acute hepatitis		3 (28)			
	**Renal**		**6 (18)**		42 (23–62)	19 (1–107)	49 (9–324)
		Acute tubular necrosis		3 (50)			
		Interstitial nephritis		5 (83)			

*IQR shown if n≥10; range shown if n<10

†Some decedents had more than one pathogenic organism identified

‡Positive viral PCR was not considered sufficient evidence of disease

CMV: cytomegalovirus; CSF: cerebrospinal fluid; IQR: interquartile range; MIA: minimally-invasive autopsy

### Infections with non-tuberculous mycobacteria and other bacteria

Of the 34 decedents, three (9%) had evidence of disease caused by non-tuberculous mycobacteria (NTM): two grew *Mycobacterium avium* and one grew *Mycobacterium intracellulare* from multiple sites. Excluding mycobacteria and organisms considered artefact, 23/34 (68%) decedents had culture evidence of pathogenic bacterial infections: 14/23 (61%) grew *Klebsiella* spp. from non-splenic samples; four (17%) grew *S*. *aureus*; two (9%) grew *Pseudomonas* spp.; and *S*. *pneumoniae*, *Salmonella* sp., *Nocardia* sp., and *Haemophilus* sp. were cultured from one (4%) decedent each. Pathogenic bacteria were most frequently isolated from BAL samples (18 positive cultures) and lung tissue (12 positive; S1 Table). Of the 33 decedents with lung tissue available for histological examination, 11 (33%) had microscopic evidence of bacterial pneumonia (Table 2), nine (82%) of whom also grew pathogenic bacteria from BAL and/or lung tissue, most frequently *Klebsiella* spp.. Of the 30 decedents with CSF samples, two (7%) were culture positive, one growing *Salmonella* sp. and the other *S*. *aureus* and *S*. *pneumoniae*.

Bacterial PCR was conducted on 13 CSF samples and 10 blood samples: *S*. *pneumoniae* was detected in 3/13 (23%) CSF samples and 1/10 (10%) blood sample; and *H*. *influenzae* was detected in 1/10 (10%) blood sample. PCR of 11 BAL specimens and 13 naso-/oropharyngeal swabs for atypical pneumonia-causing pathogens and *Bordetella* spp. and testing of seven urine samples for *Legionella pneumophila* and pneumococcal antigen yielded no positive results (S1 Table).

### Cryptococcal disease

Evidence of cryptococcal disease was found in 4/34 (12%) decedents with enrolment CD4 counts ranging from 1–56 cells/μL and time from enrolment to death 10–373 days. Three (75%) decedents had histological evidence of cryptococcal disease (Fig 1), two in more than one organ, and *Cryptococcus neoformans* was grown from two (50%) decedents, one from lungs and the other from liver and CSF. Two decedents were treated for cryptococcosis prior to death, receiving 800mg oral fluconazole for four days and 800mg intravenous fluconazole for one day, respectively (decedents 02 and 05; S2 Table).

### Other fungal and viral infections

Two of 34 (6%) decedents had evidence of cytomegalovirus (CMV) disease on histological examination, both with disseminated disease, and a further two (6%) had evidence of *Pneumocystis* pneumonia (PCP; Table 2; Fig 1). Viral PCR assays were conducted on specimens from 14/34 (41%) decedents (S1 Table). Of the 13 with BAL specimens and/or naso-/oropharyngeal swabs available for viral testing, rhinovirus was detected four (31%) times and influenza virus and respiratory syncytial virus detected once each (8%). CSF samples from 11 decedents were available for viral testing, EBV was detected eight times (73%), CMV seven times (64%); parvovirus B19 twice (18%); and influenza A virus, human-Herpes virus 7, and varicella zoster virus once each (9%).

### Decedents with multiple pathogens identified

**Fig 2:**
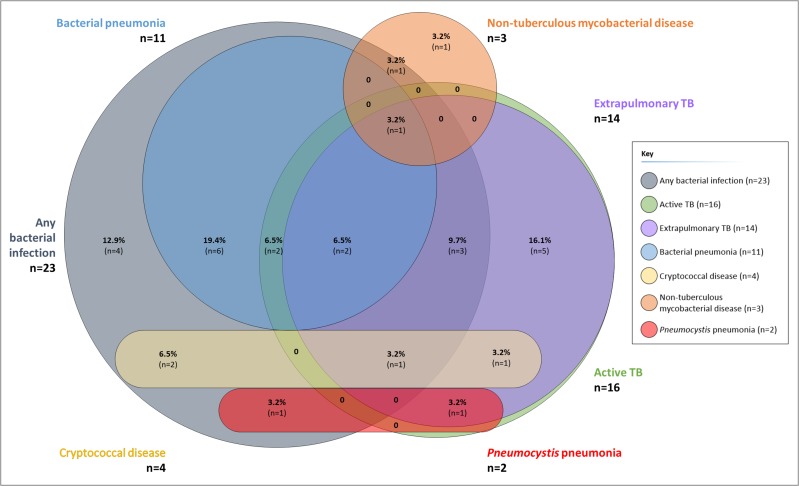
Venn diagram illustrating overlap between diagnoses made at autopsy: any active TB; extrapulmonary TB; any bacterial infection; bacterial pneumonia; disease due to non-tuberculous mycobacteria; cryptococcal disease; and *Pneumocystis* pneumonia (n = 31*). *Figure only includes decedents with autopsy evidence of disease due to specified pathogens

Twenty (59%) decedents had either histological or culture evidence of at least two infections present at the same or different sites (Fig 2). Of the 16 decedents with evidence of TB, nine (56%) had evidence of bacterial disease at one or more sites; two (13%) had evidence of cryptococcal disease; and one (6%) each had evidence of CMV disease (Fig 1), PCP, and disease due to NTM. Of the 23 decedents with evidence of bacterial infections, three (13%) also had evidence of cryptococcal disease; two (9%) disease due to CMV; two (9%) disease due to non-tuberculous mycobacteria; one (4%) due to PCP; and four (17%) due to diseases caused by other viruses.

### Additional findings

Liver tissue from 30 decedents underwent histological examination,18 (60%) showed evidence of inflammation: 5/18 (28%) had acute or chronic hepatitis, four of which were thought likely to be viral in origin (Fig 1); 12/18 (67%) had evidence of steatosis, one with non-alcoholic steato-hepatitis; and 6/18 (33%) had evidence of non-specific portal triaditis. Renal samples were also collected incidentally (nine from the right kidney, thirteen from the left): 6/13 (46%) decedents showed evidence of interstitial nephritis (n = 5) and/or acute tubular necrosis (n = 3) in one or both kidneys (Table 2).

## Discussion

To our knowledge this is the first study that has followed up a prospective cohort of HIV-positive outpatients conducting minimally invasive autopsies on those who died. This study is also unusual in that it includes a number of deaths that occurred outside hospitals, thereby including individuals not well represented in previous autopsy studies of HIV-positive individuals.

### Disease prevalence and overlap

We conducted MIA on as many TB Fast Track decedents as possible, all of whom were not taking ART or TB treatment at the time of enrolment. Those who had MIA and those who did not were largely similar with regard to characteristics at enrolment and during follow up, although those with MIA were more likely to have been treated for TB between enrolment and death. This, together with the likelihood that MIA will miss some cases that may be identified by full autopsy [18], suggests that the prevalence of TB at death among the entire population of decedents in TB Fast Track may have been even higher than the almost 50% found in this sample. Nearly 90% of those with evidence of TB had disease in at least one extrapulmonary site and 38% were not on TB treatment at the time of death. These findings, in line with data published by Omar et al. [5], suggest that TB is as frequent a pathogen among those attending primary care clinics as it is among the in-patients previously investigated in autopsy studies. Our findings are consistent with most autopsy studies done in sub-Saharan Africa over the last 20 years [4–6]. A recent systematic review [6] found the pooled prevalence of TB at autopsy in HIV-positive adults to be 39.7%, of which 87.9% was extrapulmonary and/or disseminated and 45.8% was not diagnosed before death. The review included four studies conducted during the ART era and, though overall ART coverage was not included, TB prevalence in these four studies was equal to or greater than in studies conducted prior to ART rollout, suggesting that individuals are initiating ART too late or are not linked to treatment at all. Studies conducted in clinics in South Africa have previously highlighted the large burden of undiagnosed TB among the ambulatory HIV-positive population [30,31], but investigation for TB is largely based on reporting respiratory symptoms, using sputum-based diagnostic tests [32,33]. A recent study conducted in Cape Town found that, of 139 HIV-positive individuals diagnosed with TB during admission to hospital (median CD4 count 80 cells/μL; 35% on ART for a median 1.3 years), 115 (83%) had at least one non-respiratory sample that was positive on testing with Xpert® MTB/RIF, or culture positive for *M*. *tuberculosis* [34]. Non-respiratory samples included blood, urine, and CSF. WHO recommends Xpert® MTB/RIF for use on extrapulmonary samples [35]. However, there are formidable practical and financial challenges in establishing alternative diagnostic algorithms and care pathways in primary care in resource-limited settings, in particular, the lack of a sensitive, cost-effective, point-of-care diagnostic test, and the difficulties in obtaining suitable specimens samples in individuals who cannot produce sputum. Regardless, the data suggest that these are obstacles that must be overcome in order to achieve any meaningful reduction in TB mortality.

A striking finding in our study was the high proportion of decedents with evidence of two or more infections at autopsy, sometimes at different sites, or concurrent at the same site. These are similar to results from a study conducted in 2009 among 39 HIV-positive ART-eligible adults in a hospital in South Africa [36], which attributed a third of deaths to bacterial infections, with several decedents showing evidence of multiple infections. Another study, involving full autopsy on adults (≥16 years) in a tertiary hospital in Zambia, 101 of whom were HIV-positive, found that 39/101 (39%) had autopsy evidence of ‘pyogenic pneumonia’, with 21/39 (54%) also showing evidence of active TB disease [4]. Among decedents in our study, most bacterial infections were due to common pathogens, such as *Klebsiella* spp., *Salmonella* spp., *H*. *influenzae*, and *S*. *aureus*. More needs to be done, through increased awareness among clinicians and in the design of clinical guidelines, to target and treat other opportunistic infections in those with advanced disease, even those already receiving prophylaxis, TB treatment, or ART. Longer term, initiating ART at higher CD4 counts will help reduce the likelihood of progression to this stage of advanced disease; recent guidelines advocating ART in all people living with HIV are an important step towards making this a reality [37].

### Interpretation of autopsy findings

*M*. *tuberculosis* can survive for years after the death of an infected individual and after preservation in formalin [38,39]. In our cohort, seven (64%) of the 11 individuals with *M*. *tuberculosis* culture-positive samples had received TB treatment for a median 122 (IQR 48–176) days. The interpretation of culture results from autopsy specimens is not straightforward and may be complicated further when the procedure is conducted many days after death, potentially providing greater opportunity for tissue autolysis and translocation of organisms to compartments they did not occupy in life [40–42]. However, there is evidence to show that the time from death to autopsy does not have a major effect on false-positive bacterial results if the body is kept refrigerated [43,44]. One study, conducted among 507 infants with sudden and unexpected deaths, even suggests that a longer interval may allow for fewer false-positive results [45]; and there are reports of pathogens recovered from bodies in states of advanced decomposition [46,47]. In our study, samples from all sites, except CSF, grew some organisms that were likely artefact (S1 Table); data from each decedent were reviewed individually by a microbiologist to decide on likely contaminants. This further highlights the challenges faced in assigning causes of death in these individuals, in whom many pathogens may cause disease at one time. Results of any microbiological tests, including mycobacterial, must be reviewed with clinical history and, ideally, histology in order to more accurately estimate prevalence of active disease and/or cause of death. The development of standardised guidelines to this end is suggested.

### MIA in surveillance

The consistent under-diagnosis of TB ante mortem [6] suggests that MIA may have a role in routine surveillance to estimate TB prevalence at death more accurately. If MIA was to be simplified for this purpose, our data suggest that respiratory samples (BAL and lung tissue) would provide the highest yield for TB diagnosis. Combining BAL Xpert® MTB/RIF with BAL mycobacterial culture would have detected nine of our TB cases; adding mycobacterial culture of lung tissue would add a further two; and histological examination of lung tissue a further three; accounting for 88% of the TB seen in our decedents. The role of Xpert® MTB/RIF as part of the autopsy process is not established: a recent study testing post mortem liver, lung, and brain tissue found that it detected TB in 7/8 (87.5%) cases, although the gold standard used was another PCR technique, rather than liquid culture [21]. In our study, only 5/14 (35.7%) decedents with BAL specimens and evidence of TB had a positive Xpert® MTB/RIF, though this test was done only on BAL specimens, whereas culture and histology were conducted on specimens from many other anatomical sites, many of them extrapulmonary. There were three decedents whose BAL specimens were Xpert® MTB/RIF negative, but culture positive for MTB (decedents 2, 4, and 33; S2 Table). More work is needed to establish the optimum use of this and other molecular techniques in post mortem specimens.

This study has limitations: autopsy coverage of deaths occurring among participants in TB Fast Track was low, at around 10%, but those included appear largely representative of all those who died. Although MIA provides less information than complete autopsy, particularly with regards to non-infectious diagnoses, its greater acceptability to families and participants is a major advantage; and it was not possible for one operator to conduct all 34 MIAs, leading to some variability in tissue sampling. Strengths of the study include being nested within a trial that recruited a widely representative sample of adults with advanced HIV disease at high risk of active TB; mycobacterial culture and histology being conducted on all decedents; and samples from different anatomical sites being individually tested, which may provide guidance for future MIA-based surveillance programmes.

## Conclusions

The autopsy prevalence of tuberculosis among adults with advanced HIV disease who die before initiating ART or early on ART is high and is likely a major contributor to mortality. The large proportion of individuals with autopsy evidence of potentially treatable bacterial and/or fungal infections, often in addition to TB, should be considered when assessing these high-risk patients in primary or secondary care. Evidence-based interventions are urgently needed that prioritise timely and thorough investigation for those with advanced HIV disease; allow for empiric treatment when indicated; and promote early initiation of ART.

## Supporting Information

S1 Table Samples targeted, success rates, and yield from culture and molecular tests for each specimen (n = 34). (DOCX)

S2 Table Autopsy findings for each decedent: histological, microbiological, DNA, and immunological evidence of tuberculosis, bacterial disease, and other diseases, listed by time from death to MIA (n = 34). (DOCX)
