# Supplementary material for: Autopsy Prevalence of Tuberculosis and Other Potentially Treatable Infections among Adults with Advanced HIV Enrolled in Out-Patient Care in South Africa
Source: PLoS One. 2016 Nov 9;11(11):e0166158. doi: 10.1371/journal.pone.0166158 (PMC5102350; doi:10.1371/journal.pone.0166158)
Supplement: S1 Table — (DOCX) [file pone.0166158.s001.docx]

S1 Table. Samples targeted, success rates, and yield from culture and molecular tests for each specimen (n=34)

| **Site/sample** | | | **ALL**  **n (%)** | **BAL**  **n (%)** | **Blood**  **n (%)** | **CSF**  **n (%)** | **Liver**  **n (%)** | **Lungs**  **n (%)** | **NP/OP**  **n (%)** | **Spleen**  **n (%)** | **Urine**  **n (%)** |
| --- | --- | --- | --- | --- | --- | --- | --- | --- | --- | --- | --- |
| Decedents where attempts made | | | - | 31 | 13 | 32 | 34 | 34 | 13 | 34 | 12 |
| **Successful attempts** (based on histology for tissue) | | | **-** | **30 (97)** | **10 (77)** | **30 (94)** | **30 (88)** | **33 (97)** | **13 (100)** | **21 (62)** | **7 (58)** |
| **MYCOBACTERIA** | **Mycobacterial culture** | **Tested** | **164** | **30** | **-** | **28** | **33** | **32** | **-** | **34** | **7** |
|  |  | Positive (MTB) | 26 (16) | 7 (23) |  | 5 (18) | 4 (12) | 5 (16) |  | 5 (15) | 0 |
|  |  | Positive (NTM) | 11 (7) | 1 (3) |  | 1 (4) | 3 (9) | 2 (6) |  | 3 (9) | 1 (14) |
|  |  | Negative | 113 (69) | 16 (53) |  | 20 (71) | 24 (73) | 23 (72) |  | 25 (74) | 5 (71) |
|  |  | Contaminated | 14 (9) | 6 (20) |  | 2 (7) | 2 (7) | 2 (6) |  | 1 (3) | 1 (14) |
|  | **Xpert**® **MTB/RIF** | **Tested** | **29** | **29** | **-** | **-** | **-** | **-** | **-** | **-** | **-** |
|  |  | Positive | 5 (17) | 5 (17) |  |  |  |  |  |  |  |
| **BACTERIA** | **Bacterial culture†** | **Tested** | **131** | **29** | **7** | **14** | **16** | **33** | **-** | **32** | **-** |
|  |  | Positive | 37 (28) | 18 (62) | 2 (29) | 2 (14) | 1 (6) | 12 (36) |  | 2 (6) |  |
|  |  | Negative | 45 (34) | 2 (7) | 2 (29) | 12 (86) | 9 (56) | 11 (33) |  | 9 (28) |  |
|  |  | Contaminated | 49 (37) | 9 (31) | 3 (43) | 0 | 6 (38) | 10 (30) |  | 21 (66) |  |
|  | **Multiplex PCR (meningitis)** | **Tested** | **23** | **-** | **10** | **13** | **-** | **-** | **-** | **-** | **-** |
|  |  | *H. influenzae* | 1 (4) |  | 1 (10) | 0 |  |  |  |  |  |
|  |  | *S. pneumoniae* | 3 (13) |  | 1 (10) | 2 (23) |  |  |  |  |  |
|  |  | *N. meningitidis* | 0 |  | 0 | 0 |  |  |  |  |  |
|  | **Multiplex PCR (atypical pneumonias)** | **Tested** | **24** | **11** | **-** | **-** | **-** | **-** | **13** | **-** | **-** |
|  |  | *M. pneumoniae* | 0 | 0 |  |  |  |  | 0 |  |  |
|  |  | *C. pneumoniae* | 0 | 0 |  |  |  |  | 0 |  |  |
|  |  | *Legionella spp.* | 0 | 0 |  |  |  |  | 0 |  |  |
|  | ***Bordetella* spp.** | **Tested** | **24** | **11** |  |  |  |  | **13** |  |  |
|  |  | Positive | 0 | 0 |  |  |  |  | 0 |  |  |
|  | **Urinary antigen** | **Tested** | **7** | **-** | **-** | **-** | **-** | **-** | **-** | **-** | **7** |
|  |  | *S. pneumoniae* | 0 |  |  |  |  |  |  |  | 0 |
|  |  | *L. pneumophila* | 0 |  |  |  |  |  |  |  | 0 |
| **FUNGI** | **Fungal culture** | **Tested** | **131** | **29** | **7** | **14** | **16** | **33** | **-** | **32** | **-** |
|  |  | Positive | 28 (21) | 7 (24) | 0 | 1 (7) | 2 (13) | 13 (40) |  | 5 (16) |  |
|  | **Cryptococcal antigen** | **Tested** | **33** | **-** | **7** | **26** | **-** | **-** | **-** | **-** | **-** |
|  |  | Positive | 2 (6) |  | 0 | 2 (8) |  |  |  |  |  |
|  | ***Pneumocystis* IFA** | **Tested** | **26** | **26** | **-** | **-** | **-** | **-** | **-** | **-** | **-** |
|  |  | Positive | 2 (8) | 2 (8) |  |  |  |  |  |  |  |
| **VIRUSES** | **Multiplex PCR (respiratory)** | **Tested** | **24** | **11** | **-** | **-** | **-** | **-** | **13** | **-** | **-** |
|  |  | Para-influenza viruses 1-3 | 0 | 0 |  |  |  |  | 0 |  |  |
|  |  | Adenovirus | 0 | 0 |  |  |  |  | 0 |  |  |
|  |  | Enterovirus | 0 | 0 |  |  |  |  | 0 |  |  |
|  |  | Human metapneumovirus | 0 | 0 |  |  |  |  | 0 |  |  |
|  |  | Respiratory syncytial virus | 1 (4) | 0 |  |  |  |  | 1 (8) |  |  |
|  |  | Rhinovirus | 8 (33) | 4 (36) |  |  |  |  | 4 (31) |  |  |
|  |  | Influenza A virus | 1 (4) | 0 |  |  |  |  | 1 (8) |  |  |
|  |  | Influenza B virus | 0 | 0 |  |  |  |  | 0 |  |  |
|  | **Multiplex PCR (neurological)** | **Tested** | **11** | **-** | **-** | **11** | **-** | **-** | **-** | **-** | **-** |
|  |  | Cytomegalovirus | 7 (64) |  |  | 7 (64) |  |  |  |  |  |
|  |  | Epstein-Barr virus | 8 (73) |  |  | 8 (73) |  |  |  |  |  |
|  |  | Herpes simplex virus 1 & 2 | 0 |  |  | 0 |  |  |  |  |  |
|  |  | Human herpes virus 6 | 0 |  |  | 0 |  |  |  |  |  |
|  |  | Human herpes virus 7 | 1 (9) |  |  | 1 (9) |  |  |  |  |  |
|  |  | Parechoviruses | 0 |  |  | 0 |  |  |  |  |  |
|  |  | Parvovirus B19 | 2 (18) |  |  | 2 (18) |  |  |  |  |  |
|  |  | Varicella zoster virus | 1 (9) |  |  | 1 (9) |  |  |  |  |  |
| †A non-splenic sample growing only *Enterococcus* spp., and/or *Enterobacter* spp., and/or *E. coli*, and/or *Proteus* spp., and/or *Bacillus* spp., and/or coagulase negative staphylococci, and/or *Viridans streptococci* was considered contaminated, as was a splenic sample growing only one of the above, or growing only Gram-negative organisms in the absence of the growth of the same organism from another site.  BAL: bronchoalveolar lavage; CSF: cerebrospinal fluid; IFA: indirect fluorescent antibody; MTB: *M. tuberculosis*; NP/OP: naso-/oro-pharyngeal swabs; NTM: non-tuberculous mycobacteria; PCR: polymerase-chain reaction | | | | | | | | | | | |
|  | | | | | | | | | | | |
