# Supplementary material for: Autopsy Prevalence of Tuberculosis and Other Potentially Treatable Infections among Adults with Advanced HIV Enrolled in Out-Patient Care in South Africa
Source: PLoS One. 2016 Nov 9;11(11):e0166158. doi: 10.1371/journal.pone.0166158 (PMC5102350; doi:10.1371/journal.pone.0166158)
Supplement: S2 Table — (DOCX) [file pone.0166158.s002.docx]

S2 Table. Autopsy findings for each participant: histological, microbiological, DNA, and immunological evidence of tuberculosis, bacterial disease, and other diseases, listed by time from death to MIA (n=34)

| **ID*** | **Time in days** | | | | **Evidence of tuberculosis** | | | | **Evidence of disease due to NTM and/or other bacteria** | | **Other histological, microbiological,**  **DNA, and immunological findings**  **and site(s)** |
| --- | --- | --- | --- | --- | --- | --- | --- | --- | --- | --- | --- |
|  | **From enrolment**  **to death** | **From death**  **to MIA** | **On TB Tx** | **On ART** | **Histological evidence**  **and**  **site(s)** | **Positive culture**  **and**  **site(s)** | **Xpert**® **MTB/RIF positive (BAL only)** | **If MTB found, INH/RIF sensitive?** | **Histological evidence**  **and site(s)** | **Organism(s) grown**  **or detected by PCR**  **and site(s)** |  |
| **07** | 48 | 1 | 48 | - | - | CSF, LIV, SPL | - | ✓ | **-** | *Klebsiella* spp. (BAL) | **CrAg positive** (CSF) |
| **30** | 1 | 2 | - | - | - | - | - | **-** | LL, RL | *H. parainfluenzae* (lungs) | *-* |
| **06** | 21 | 2 | - | 15 | - | - | - | **-** | RL | *Pseudomonas* spp. (BAL, lungs) | **Histology:** Extensive autolysis (LK, RK) |
| **15** | 34 | 2 | - | 30 | - | - | - | - | - | - | **Histology:** Pneumonitis, ?viral + organising hyaline membrane disease (LL, RL) |
| **31** | 63 | 2 | - | 56 | LIV, LL, RL, SPL | - | ✓ | RIF resistant (BAL) | **-** | **-** | **Histology:** Extramedullary haematopoiesis (RL; LL)  **PCR:** EBV (CSF); PVB19 (CSF) |
| **08** | 28 | 3 | - | - | LIV, LL, RL | BAL, LIV, lungs | ✓ | ✓ | RL | *K. pneumoniae* (BAL; lungs) | **Histology:** Florid superadded CMV infection (LIV; LL; RL) |
| **24** | 33 | 3 | 33 | 13 | LIV, SPL | - | - | - | LL, RL | *M. avium* (LIV, SPL) | **PCR:** CMV (CSF); EBV (CSF) |
| **09** | 38 | 3 | - | 29 | LL, LK | - | - | **-** | **-** | *K. pneumoniae* (BAL; lungs) | **Histology:** Acute tubular necrosis with underlying acute suppurative pyelonephritis (RK; LK) |
| **10** | 41 | 3 | 48 | 32 | RL | - | **-** | **-** | RL | *Klebsiella* spp. (BAL) | **Histology:** Acute tubular injury (RK; LK) |
| **18** | 49 | 3 | - | 50 | LIV, LL, RL, SPL | BAL, LIV, lungs, SPL | ✓ | RIF resistant (BAL) | - | - | *-* |
| **33** | 182 | 3 | 182 | 148 | - | BAL, CSF | - | ✓ | - | - | **Histology:** Severe PCP (LL; RL)  **PCR:** *H. influenzae* (BLD); Rhinovirus (BAL; NP/OP) |
| **12** | 9 | 4 | 9 | - | - | - | - | **-** | RL | *-* | *-* |
| **02** | 10 | 4 | - | 10 | - | BAL, CSF | **-** | ✓ | - | - | **Histology:** Disseminated cryptococcosis (LIV; LL; RL)  **Culture:** *C. neoformans* (lungs)  **CrAg positive** (CSF) |
| **34** | 184 | 4 | 195 | 74 | - | - | - | **-** |  | *K. pneumoniae* (BAL, BLD, lungs) | **Histology:** Disseminated CMV infection (LIV; LL; RL); Chronic active hepatitis B infection (LIV) |
| **29** | 359 | 4 | 40 | 88 | LL, RL | - | **-** | **-** | LL, RL | *Nocardia* sp. (lungs);  *K. pneumoniae* (BAL; lungs) | **PCR:** CMV (CSF); Rhinovirus (BAL; NP/OP) |
| **13** | 14 | 5 | 14 | - | - | - | - | **-** | RL | *P. aeruginosa* (BAL);  *K pneumoniae* (BAL) | **Histology:** Interstitial pneumonitis, ?viral (LL) |
| **20** | 62 | 5 | - | 28 | LIV, LL,  RL, SPL | BAL, CSF, LIV,  lungs, SPL | ✓ | ✓ | **-** | *S. pneumoniae* 15A/F (CSF);  *Klebsiella* spp. (BAL) | **PCR:** Respiratory syncytial virus (NP/OP) |
| **32** | 100 | 5 | - | 100 | - | - | - | - | Soft tissue | *Salmonella* spp. (BAL, BLD, CSF, LIV, lungs, SPL)  *S. aureus* (BAL, BLD)  *H. influenzae* (BLD) | **Histology:** Pneumonitis, ?viral (LL; RL)  **PCR:** CMV (CSF); EBV (CSF); PVB19 (CSF) |
| **19** | 373 | 5 | 329 | 90 | - | - | - | **-** | **-** | *S. aureus* (BAL);  *K. pneumoniae* (BAL) | **Histology:** Disseminated cryptococcosis (LIV**,** LL, RL, SPL)  **Culture:** *C. neoformans* (CSF, LIV)  **CrAg positive** (CSF)  **PCR:** Rhinovirus (BAL, NP/OP); CMV (CSF); EBV (CSF) |
| **16** | 4 | 6 | 4 | - | - | - | - | - | *-* | *-* | *-* |
| **26** | 8 | 6 | 7 | - | - | - | - | **-** | **-** | *S. pneumoniae* 19F (BLD; CSF) | **PCR:** EBV (CSF) |
| **03** | 82 | 6 | 19 | 66 | LIV, SPL | SPL | ✓ | ✓ | *-* | *-* | **Histology:** Significant steatosis (LIV) |
| **14** | 128 | 6 | 63 | 126 | LIV, RL | BAL, CSF | **-** | ✓ | LL | *K. pneumoniae* (BAL, lungs) | - |
| **27** | 324 | 6 | - | 317 | - | - | - | - | LIV | *M. intracellulare* (LIV; lungs; SPL)  *K. pneumoniae* (lungs) | **Histology:** Acute tubular necrosis (LK) |
| **28** | 450 | 6 | 71 | 422 | LIV, LL, RL, SPL | Lungs | - | ✓ | - | - | **PCR:** EBV (CSF) |
| **05** | 57 | 7 | 57 | - | - | - | - | **-** | **-** | *K. pneumoniae* (lungs) | **Histology:** Severe cryptococcal pneumonia (LL, RL)  **Culture:** *Aspergillus fumigatus* (lungs) |
| **22** | 285 | 7 | - | 284 | - | - | - | **-** | LL, RL | *S. aureus* (BAL; CSF; lungs; SPL);  *S. pneumoniae* 9A/V, 1 (CSF) | **PCR:** Influenza A virus H32N (CSF, NP/OP); CMV (CSF) EBV (CSF); HHV-7 (CSF) |
| **17** | 14 | 8 | - | - | - | - | - | - | *-* | *S. aureus* (lungs);  *P. aeruginosa* (BAL); | **Histology:** Profound non-alcoholic steato-hepatitis |
| **23** | 21 | 8 | - | 21 | - | - | - | **-** | LIV, LL, RL, SPL | *M. avium*  (BAL; CSF; LIV; lungs; SPL; urine) | **PCR:** CMV (CSF); VZV (CSF) |
| **04** | 122 | 8 | 120 | 108 | LIV, LL, pleura, RL | BAL | - | ✓ | **-** | *-* | - |
| **25**† | 122 | 8 | 126 | 46 | - | - | - | **-** | **-** | **-** | **Histology:** mild autolytic changes (SPL) |
| **21** | 304 | 8 | 304 | 276 | - | - | - | **-** | LL | *Klebsiella* spp. (BAL, lungs) | **Histology:** Chronic hepatitis  **PCR:** Rhinovirus (BAL, NP/OP); CMV (CSF); EBV (CSF) |
| **01** | 175 | 9 | 173 | 153 | - | SPL | - | ✓ | **-** | *Klebsiella spp.* (BAL) | **Histology:** Non-specific portal triaditis (LIV) |
| **11** | 84 | 11 | 82 | 67 | - | - | - | **-** | **-** | *K. pneumoniae* (BAL) | **Histology:** Severe bilateral PCP  **PCP immunofluorescence positive** (BAL) |
| *ID denotes chronological order in which MIA was conducted (first October 2013, last June 2015) †Samples obtained as part of complete autopsy – heavily contaminated  BAL: broncheo-alveolar lavage; BLD: blood; CMV: Cytomegalovirus; CSF: cerebrospinal fluid; EBV: Epstein-Barr virus; HHV: Human Herpes virus; INH: Isoniazid; LIV: liver; LK: left kidney; LL: left lung; MIA: minimally-invasive autopsy; MTB: *M. tuberculosis*; NP/OP: nasopharyngeal/oropharyngeal swab; NTM: non-tuberculous mycobacteria; PCP: *Pneumocystis* pneumonia; PCR: polymerase chain reaction; PVB19: Parvovirus B19; RIF: Rifampicin; RK: right kidney; RL: right lung; SPL: spleen; TB: tuberculosis; Tx: treatment; VZV: Varicella zoster virus | | | | | | | | | | | |
